# Urinary Retention Evaluation and Catheterization Algorithm for Adult Inpatients

**DOI:** 10.1001/jamanetworkopen.2024.22281

**Published:** 2024-07-16

**Authors:** Kristin Chrouser, Karen E. Fowler, Jason D. Mann, Martha Quinn, Jessica Ameling, Samantha Hendren, Greta Krapohl, Ted A. Skolarus, Steven J. Bernstein, Jennifer Meddings

**Affiliations:** 1 Department of Urology, University of Michigan, Ann Arbor; 2 VA Center for Clinical Management Research, VA Ann Arbor Healthcare System, Ann Arbor, Michigan; 3 Division of Geriatrics, Department of Internal Medicine, University of Michigan, Ann Arbor; 4 School of Public Health, University of Michigan, Ann Arbor; 5 Division of General Medicine, Department of Internal Medicine, University of Michigan, Ann Arbor; 6 Department of Surgery, University of Michigan, Ann Arbor; 7 Michigan Surgical Quality Collaborative, Ann Arbor; 8 Veterans Health Administration National Center for Patient Safety, Ann Arbor, Michigan; 9 Department of Surgery, University of Chicago, Chicago, Illinois; 10 Division of General Pediatrics, Department of Pediatrics and Communicable Diseases, University of Michigan, Ann Arbor

## Abstract

**Importance:**

Acute urinary retention (UR) is common, yet variations in diagnosis and management can lead to inappropriate catheterization and harm.

**Objective:**

To develop an algorithm for screening and management of UR among adult inpatients.

**Design, Setting, and Participants:**

In this mixed-methods study using the RAND/UCLA Appropriateness Method and qualitative interviews, an 11-member multidisciplinary expert panel of nurses and physicians from across the US used a formal multi-round process from March to May 2015 to rate 107 clinical scenarios involving diagnosis and management of adult UR in postoperative and medical inpatients. The panel ratings informed the first algorithm draft. Semistructured interviews were conducted from October 2020 to May 2021 with 33 frontline clinicians—nurses and surgeons from 5 Michigan hospitals—to gather feedback and inform algorithm refinements.

**Main Outcomes and Measures:**

Panelists categorized scenarios assessing when to use bladder scanners, catheterization at various scanned bladder volumes, and choice of catheterization modalities as appropriate, inappropriate, or uncertain. Next, qualitative methods were used to understand the perceived need, usability, and potential algorithm uses.

**Results:**

The 11-member expert panel (10 men and 1 woman) used the RAND/UCLA Appropriateness Method to develop a UR algorithm including the following: (1) bladder scanners were preferred over catheterization for UR diagnosis in symptomatic patients or starting as soon as 3 hours since last void if asymptomatic, (2) bladder scanner volumes appropriate to prompt catheterization were 300 mL or greater in symptomatic patients and 500 mL or greater in asymptomatic patients, and (3) intermittent was preferred to indwelling catheterization for managing lower bladder volumes. Interview findings were organized into 3 domains (perceived need, feedback on algorithm, and implementation suggestions). The 33 frontline clinicians (9 men and 24 women) who reviewed the algorithm reported that an evidence-based protocol (1) was needed and could be helpful to clinicians, (2) should be simple and graphically appealing to improve rapid clinician review, and (3) should be integrated within the electronic medical record and prominently displayed in hospital units to increase awareness. The draft algorithm was iteratively refined based on stakeholder feedback.

**Conclusions and Relevance:**

In this study using a systematic, multidisciplinary, evidence- and expert opinion–based approach, a UR evaluation and catheterization algorithm was developed to improve patient safety by increasing appropriate use of bladder scanners and catheterization. This algorithm addresses the need for practical guidance to manage UR among adult inpatients.

## Key Points

**Question:**

How should urinary retention (UR) be managed in the inpatient setting?

**Findings:**

In this mixed-methods study using the RAND/UCLA Appropriateness Method and qualitative interviews, an algorithm for screening and management of UR among adult inpatients was developed. The algorithm included guidance on when to bladder scan, what bladder scanner volumes should prompt catheterization, and when intermittent catheterization is preferred to indwelling catheterization.

**Meaning:**

The resulting algorithm was a 1-page practical guidance document to manage UR among inpatients that can be readily adopted by hospitals.

## Teaser

This mixed-methods study uses the RAND/UCLA Appropriateness Method and qualitative interviews with expert clinicians to develop an algorithm for screening and management of adult inpatients with urinary retention.

## Introduction

Urinary catheters are commonly used in hospitalized medical and surgical patients. However, there are no guidelines or widely accepted algorithms for screening and management of urinary retention (UR) in these patients. Balancing the potential complications of untreated UR (eg, pain, bladder dysfunction) against those of urinary catheterization (eg, urethral injury, urinary tract infection) is necessary when deciding whether to insert a catheter.^1,2,3,4,5,6^

Although UR algorithms exist for outpatient populations,^7,8^ there is limited evidence to guide management in hospitalized patients. Outpatient surgical populations are generally healthier and undergo less-invasive procedures than those requiring inpatient care,^9^ limiting the generalizability of outpatient recommendations. Algorithms for outpatients use symptoms and risk stratification (eg, hydronephrosis, recurrent infections) to guide catheterization recommendations instead of postvoid residual volumes commonly used in hospitals.^7^ Inpatients may experience evolving disease processes and changing medications and fluid volumes that acutely impact bladder function and voiding. To improve the quality and consistency of care for hospitalized adult medical and surgical patients with UR, we aimed to use a systematic, multidisciplinary approach based on evidence, expert opinion, and feedback from frontline medical professionals to develop and refine a urinary retention evaluation and catheterization algorithm (URECA).

## Methods

In this mixed-methods study, we applied a multimethod approach, illustrated in eFigure 1 in Supplement 1. The qualitative portion of this study was approved by the institutional review board at the University of Michigan Medical School, and oral informed consent was obtained. Panelists and interviewees were compensated for their time. This study followed the Accurate Consensus Reporting Document (ACCORD) guidelines for reporting consensus methods in biomedicine.^10^

### RAND/UCLA Appropriateness Method

We developed guidance for algorithm development using the RAND/UCLA Appropriateness Method.^11^ Our group previously used this approach to define appropriateness of urinary catheterization in hospitalized medical and postoperative general, urologic, and orthopedic surgery patients.^8,9,12^ Our group’s prior articles^8,9,12^ include detailed methods and documentation of the rating process. These methods combine scientific evidence with clinical judgment from multidisciplinary and multispecialty content experts to produce guidance regarding a procedure’s appropriateness while addressing both patient symptoms and clinical context. It has been used to define appropriate care and design quality indicators in various fields^13,14,15^ and provides useful clinical guidance when insufficient high-level evidence is available.

#### Literature Review

The RAND/UCLA Appropriateness Method begins by identifying published literature and categorizing it by relevance and level of evidence. Between September 2014 and February 2015, we searched relevant databases (ie, Web of Science, CINAHL, Embase, Cochrane, and PubMed/MEDLINE) for studies assessing bladder scanner technology, algorithms as part of UR interventions, and literature reviews involving bladder scanners. We identified and reviewed 50 unique articles meeting abstract, title, and keyword criteria, and based on content review, we narrowed the list to 26 articles (listed in eTable 1 in Supplement 1) to provide to panelists. We also developed clinical scenarios based on the literature review and piloted them with colleagues from the Patient Safety Enhancement Program at the University of Michigan and the Ann Arbor Veterans Affairs Healthcare System,^16^ along with local urogynecologists.

#### Clinician Panel Selection and Rating Process

We recruited 11 practicing clinicians experienced in evaluating and managing UR in medical and postoperative hospitalized patients. We sampled different practice types (academic, private, and government), US regions, and relevant clinical expertise. Panelists (eTable 2 in Supplement 1) included urologists, urologic nurses, and an infectious diseases physician with expertise in urinary infections.

In round 1, participants completed independent ratings of clinical scenarios. We asked panelists to rate appropriateness of each intervention (“appropriate” was defined as when expected health benefits exceeded expected negative consequences, exclusive of cost^17^) on a scale of 1 to 9, with 1 indicating harms outweigh benefits (ie, inappropriate) and 9 indicating benefits outweigh harms (ie, appropriate). A middle rating (5) indicated the benefits or harms were equivalent or the panelist could not make an informed decision. All scenarios involved adults in medical inpatient and postoperative surgical settings. We instructed panelists to use their clinical judgment and the evidence provided in the literature review, making no other assumptions.

After round 1, we held a conference call with panelists to clarify the clinical scenarios while attempting to reduce disagreement and uncertainty. Panelists met in person for round 2, during which they discussed each scenario, preliminary scores, and rating differences. Then they independently rerated each clinical scenario for a total of 107 clinical scenarios involving diagnosis and management of UR in postoperative or medical inpatients. Panelists also rated an additional 11 scenarios involving potential practices to prevent UR. Median round 2 scores determined the final classification of each scenario as appropriate (median score, 7-9), uncertain (4-6), or inappropriate (1-3). If 4 or more panelists rated a scenario as appropriate while 4 or more rated the same scenario as inappropriate, we rated the scenario as uncertain. The entire rating process took place from March to May 2015.

We assembled draft 1 of the URECA (eFigure 2 in Supplement 1) using results from the panel. The research team felt this draft had limited clinical utility since the flowsheet included large ranges of appropriateness and no guidance was offered for scenarios rated as uncertain appropriateness. Therefore, we revised the protocol by selecting cutoffs consistent with clinical practice at our institution that were within the ranges of acceptability according to the panel (draft 2 in eFigure 2 in Supplement 1). We next solicited informal expert feedback on draft 2 from 2 multidisciplinary groups, including the Surgical Urinary Catheter Care Enhancement Safety Study (SUCCESS)^18^ stakeholder committee and the Patient Safety Enhancement Program from the University of Michigan and Ann Arbor Veterans Affairs Healthcare System.^16^ These groups included experts in urinary catheter use, complication prevention, clinical education, and communication. Feedback included that the algorithm needed to be easier to read quickly by clinicians, needed to be adapted for use by nurses caring for both postoperative and medical patients, and would benefit from broader qualitative evaluation.

### Qualitative Methods to Refine the URECA

To better understand the need for and potential uses of the URECA and to solicit feedback to refine our draft algorithm, we conducted qualitative interviews with clinicians who diagnosed and managed adult postoperative UR. Interviews were part of a larger study, SUCCESS, aimed at improving safe and appropriate use of urinary catheters in surgical patients. Purposive sampling was used to include a subset of hospitals from the Michigan Surgical Quality Collaborative^19^ that varied in size, geographic location, and health system. Champions of the Michigan Surgical Quality Collaborative at each of these hospitals helped identify individuals with varied roles to interview. A total of 37 individuals agreed to be interviewed, but due to scheduling constraints, only 33 participated. Interviews took place between October 2020 and May 2021 and were conducted via videoconferencing software. We used a pilot-tested, semistructured, role-specific interview guide. The questions most relevant to this study are listed in eTable 3 in Supplement 1.

Interviews were conducted sequentially, with revisions made to the proposed algorithm after each interview based on feedback. The next interviewee would be given the updated algorithm to review. Interviews were led by a trained qualitative interviewer and clinician expert (M.Q., S.H., J.M.) and were audiorecorded, transcribed, and verified for accuracy. We used a rapid analysis approach^20,21^ to quickly integrate stakeholder feedback into the algorithm. Two team members (M.Q., J.A.) constructed a template reflecting key domains of interest (ie, perceived need for an algorithm; feedback on the current algorithm, including likes, dislikes, and suggestions for improvement; and implementation suggestions). They both read the same transcript and summarized data for each domain, including supporting quotes. Next, they compared templates, discussed discrepancies, attained consensus, and revised the template accordingly. They repeated this process with 2 more transcripts to ensure that the template was comprehensive. They then reviewed the remaining transcripts, with 1 team member conducting a primary review and the other doing a secondary review to ensure that all data were consistently and accurately captured. Once all templates were complete, we reviewed each domain for main findings and summarized with supporting quotes.

## Results

### RAND/UCLA Appropriateness Method

#### Diagnosing UR

The expert 11-member panel (10 men and 1 woman) reviewed and discussed the 107 clinical scenarios. Table 1 and eTable 4 in Supplement 1 detail panel responses to the question of bladder scanning after urinary catheter removal in postoperative or medical patients (male or female) who have no urine output for varying lengths of time and with varying symptoms (eg, pain associated with the inability to void). The panel deemed it appropriate to evaluate any symptomatic patients using a bladder scanner. Conversely, using catheterization to evaluate symptomatic or asymptomatic patients was deemed to never be appropriate if a bladder scanner is available.

**Table 1.** Appropriateness Ratings for Monitoring of Urinary Retention After Catheter Removal^a^

Time of no urine output, h	Evaluation					
	Using bladder scanner		Using ISC instead of scanning		Using IUC placement instead of scanning	
	With symptoms	Without symptoms	With symptoms	Without symptoms	With symptoms	Without symptoms
<1	Appropriate	Inappropriate	Inappropriate	Inappropriate	Inappropriate	Inappropriate
1 to <2	Appropriate	Inappropriate	Inappropriate	Inappropriate	Inappropriate	Inappropriate
2 to <3	Appropriate	Uncertain	Inappropriate	Inappropriate	Inappropriate	Inappropriate
3 to <4	Appropriate	Appropriate	Inappropriate	Inappropriate	Inappropriate	Inappropriate
4 to <5	Appropriate	Appropriate	Inappropriate	Inappropriate	Inappropriate	Inappropriate
5 to <6	Appropriate	Appropriate	Inappropriate	Inappropriate	Inappropriate	Inappropriate
>6	Appropriate	Appropriate	Inappropriate	Inappropriate	Inappropriate	Inappropriate

Abbreviations: ISC, intermittent straight catheter; IUC, indwelling urinary catheter.

^a^ Clinical scenario: lack of urine output in postoperative patient or medical inpatient. Assumption: bladder scanner is available and staff are trained to use it.

When working without bladder scanners, panel members indicated the appropriateness of an intermittent straight catheter (ISC) or indwelling urinary catheter (IUC), which varied by symptom presence and time since last urine output (eTable 5 in Supplement 1). Panelists consistently recommended ISC sooner than IUC, and longer wait times were considered appropriate in asymptomatic patients compared with symptomatic patients. We noted more uncertainty among panel members for the scenarios without bladder scanners than those with bladder scanners.

#### Bladder Scanner Volumes to Catheterize

When bladder scanners are available, bladder volume can guide the need for treatment of UR (Table 2 and eTable 6 in Supplement 1). Panel members determined that treatment was appropriate at lower volumes for symptomatic retention than for asymptomatic retention and that in symptomatic patients, use of ISC was appropriate at lower volumes than was use of IUC. In asymptomatic patients with bladder volumes of 500 mL or greater, ISC or IUC was considered appropriate, while any catheterization was considered inappropriate for volumes less than 400 mL.

**Table 2.** Appropriateness Ratings for Treatment of Urinary Retention Based on Bladder Scan Volume^a^

Bladder scan volume, mL	ISC		IUC placement overnight	
	With symptoms	Without symptoms	With symptoms	Without symptoms
<100	Inappropriate	Inappropriate	Inappropriate	Inappropriate
100-199	Uncertain	Inappropriate	Inappropriate	Inappropriate
200-299	Uncertain	Inappropriate	Inappropriate	Inappropriate
300-399	Appropriate	Inappropriate	Uncertain	Inappropriate
400-499	Appropriate	Uncertain	Appropriate	Uncertain
≥500	Appropriate	Appropriate	Appropriate	Appropriate

Abbreviations: ISC, intermittent straight catheter; IUC, indwelling urinary catheter.

^a^ Clinical scenario: postvoid scanned volumes.

#### Transitioning From ISC to IUC

Panelists felt that transitioning patients from ISC to IUC based on patient request could be appropriate depending on the clinical scenario (Table 3 and eTable 7 in Supplement 1). Patients needing ISC more than every 4 hours or whose output was more than 500 mL every 4 hours could also be appropriately transitioned to IUC. The panelists considered it inappropriate to transition to IUC for initial ISC volumes less than 500 mL, for ISC frequencies less than 5 times in 24 hours, or when needed for more than 2 days. The panel disagreed on what volume of initial retention warranted IUC instead of ISC.

**Table 3.** Appropriateness Ratings for Transitioning From ISC to IUC for Urinary Retention

Clinical scenario	Transition from ISC to IUC^a^
Timing of patient request for indwelling catheter	
Before any ISC attempt	Appropriate
After 1 ISC	Appropriate
After ≥2 ISCs	Appropriate
Frequency of need for ISC	
Once daily for ≥2 d	Inappropriate
2 Times in 24 h	Inappropriate
3 Times in 24 h	Inappropriate
4 Times in 24 h	Inappropriate
Twice daily for ≥2 d	Inappropriate
More than twice daily for ≥2 d	Inappropriate
More than every 4 h	Appropriate
ISC output, mL	
>500 Every 4 h	Appropriate
Initial <500	Inappropriate
Initial >500	Uncertain

Abbreviations: ISC, intermittent straight catheter; IUC, indwelling urinary catheter.

^a^ Assumption: Patient has no contraindication to intermittent catheterization.

In general, panelists were either uncertain or felt that it was inappropriate to perform assessments or treatments to prevent development of UR in specific patients, such as males with a history of UR. Findings are shown in eTable 8 in Supplement 1.

### Qualitative Interviews and Feedback to Refine Algorithm Development

After interviewing 33 frontline clinicians from 5 hospitals (9 men and 24 women; 10 surgical nurses, 8 surgeons, 8 nurse educators or infection preventionists, 4 urologists, and 3 surgery residents), we organized findings into 3 domains. These included (1) perceived need for a retention management algorithm; (2) feedback on the draft retention algorithm, including likes and dislikes and recommended changes; and (3) suggestions for implementation.

#### Perceived Need

Participants reported the need for an evidence-based retention algorithm in their hospital:

We found out that this is a big gap in our nursing care and we can help fill this void by establishing this protocol… and [nursing] staff like algorithms like this, they’re so easy for them to follow. (Nurse No. 113)

Furthermore, clinicians noted that postoperative UR management, including decisions regarding when to reinsert IUCs, varied considerably between units and among surgeons and that a written, evidence-based algorithm could provide consistency between units and a systematic approach to decision-making:

There are some surgeons who, a patient retains once, they will never get their Foley out. There are others who will just keep trying. It’s all over the map. I agree. It should be streamlined. (Surgeon No. 130)

Interviewees reported that an algorithm would most help clinicians who are in training (eg, residents and interns) or new to their hospital. Participants felt that these algorithms would be used and appreciated most by nurses and that physicians may be more resistant to following algorithms.

#### Feedback on Algorithm

Participants wanted a short, clear algorithm that was easy to follow. They found that the visual aspects (ie, use of arrows, boxes, and color coding) were appealing and aided quick navigation. Participants stressed the importance of limiting the amount of text:

I like how it’s clear and it’s color coded a little bit so it helps. There’s not a lot on it so you can’t get lost…it’s self-explanatory. (Nurse No. 126)

Additionally, participants liked that the algorithm prioritized patient factors, such as symptom assessment. They agreed with including language to clearly direct clinicians to begin with using ISC prior to reinserting an IUC when patients needed additional catheterization.

The most frequent critique of the earliest draft algorithm was its use of numerical ranges of urinary output (eg, 100-500 mL). Participants preferred having specific cutoff volumes (eg, 300 mL) with specific steps to take at each cutoff. Recommended changes included switching the algorithm from landscape to portrait orientation; adding specific action points, including when to call the surgeon or urologist; and adding patient-specific criteria (eg, men aged >55 years, patients with spinal cord injuries). Suggestions from frontline clinicians were iteratively incorporated into the final URECA (Figure and eFigure 3 in Supplement 1).

**Figure.**
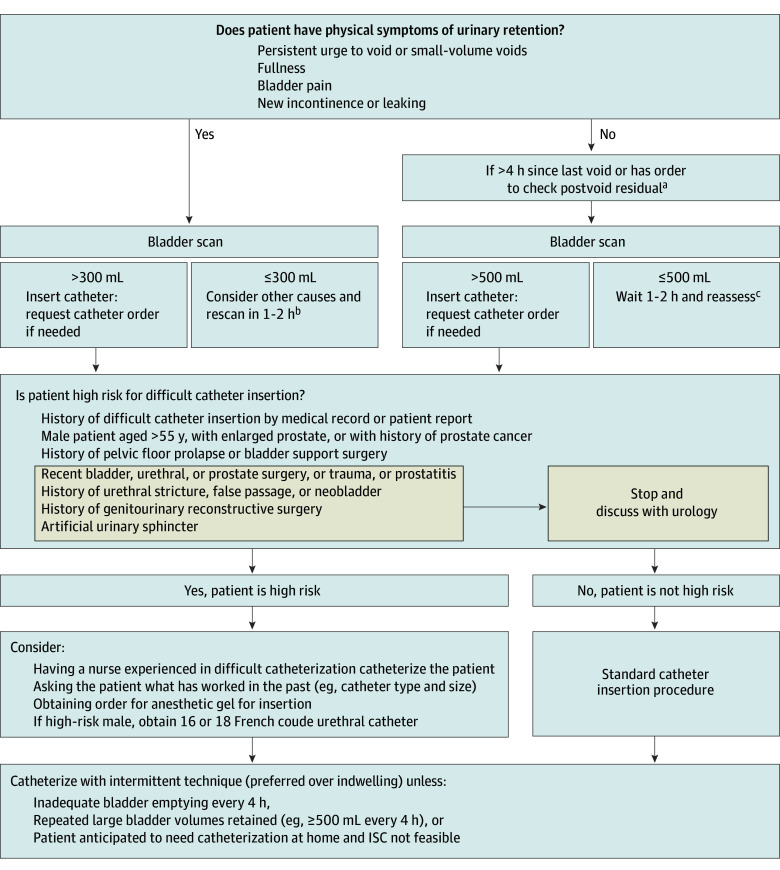
Final Evaluation and Catheterization Algorithm to Manage Urinary Retention Among Inpatients The algorithm is intended for the inpatient setting. Cutoffs were determined based on a combination of literature review, expert opinion, and local practice patterns. Of note, use of external catheters to treat urinary retention is inappropriate as external catheters only collect spontaneously voided urine. ISC indicates intermittent straight catheter. ^a^Consider checking sooner if the patient is receiving high volumes of intravenous fluid or diuretics. ^b^Other common causes of these urinary symptoms include urinary tract infection, overactive bladder, small bladder capacity, or recent catheterization. Consider contacting attending physician for further evaluation. ^c^Evaluate the patient’s fluid intake and consider increasing fluids. Call attending physician if urine output is less than 35 mL per hour, raising concern for oliguria from hypovolemia or acute kidney injury.

#### Implementation

Participants suggested that successful implementation of an algorithm would be facilitated by electronic medical record system integration and hard copies for patient rooms, nurse binders, or affixing to bladder scanners. One participant encouraged multiple formats for education since people learn in different ways. Easy access was reported as a key factor:

It only works if the protocol is available readily. If you have to spend 15 minutes looking for a protocol, nobody is going to look for it. (Surgeon No. 106)

Others suggested increasing general awareness of the algorithm, developing clinician orientation materials prior to rollout, identifying a clinician champion to facilitate implementation, and embedding the algorithm within hospital policies.

### Final Algorithm Development

The findings from the panel and the interviews as described were used to finalize the URECA (Figure and eFigure 3 in Supplement 1). The algorithm begins by reminding clinicians to assess whether the patient has any UR symptoms and, next, provides clear criteria for when bladder scanner use is appropriate. Bladder scanner volumes of more than 300 mL for symptomatic patients and more than 500 mL for asymptomatic patients are provided as thresholds for deciding to insert a catheter, along with guidance for monitoring patients not meeting these thresholds. Emphasized in the middle of the flowchart are reminders of some key risk factors for difficult insertion to assess when additional precautions may be warranted. For example, urology consultation is recommended before attempting catheterization of very high-risk patients, such as those with artificial sphincters. For patients at high risk, such as men older than 55 years or with prostatic enlargement, catheterization recommendations, such as administration by experienced nurses and the safest catheter type and size, are provided. Lastly, there is guidance for when ISC is preferred over IUC. Footnotes provide additional tips for clinicians, such as increasing awareness of hypovolemia as a cause of unexpected low urine volumes and other causes of UR symptoms, such as urinary tract infection, overactive bladder, and recent catheterization.

## Discussion

We applied a mixed-methods approach including the RAND/UCLA Appropriateness Method and qualitative methods to develop a practical algorithm based on the available evidence to assess and manage UR in postoperative and medical inpatients. We found that a combination of clinical symptoms and scanned bladder volumes could guide management and prevent unnecessary catheterization or recatheterization. Decreasing catheterization minimizes patient discomfort and reduces the risk of urethral injury and urinary tract infection. Given the lack of guidelines for UR management in this population, our robust, expert panel approach provided a framework for consistent, quality care for patients with UR.

Our study adds to the literature by providing practical guidance on assessing and managing UR based on available evidence augmented by expert opinion. Our results concur with those obtained by another multidisciplinary panel that assessed appropriate indications for initial catheter placement in hospitalized medical patients in which UR was considered an appropriate indication for catheter placement.^8^ Generally, bladder scanner accuracy is adequate for clinical purposes^22^; however, some conditions can interfere with obtaining objective data from bladder scanners used for decisions within the algorithm, such as ascites (leading to erroneous volume assessment by ultrasonography) or very low urine output due to kidney failure (extending the time needed to fill the bladder).^23^ Algorithm implementation must consider clinical context to accommodate patient variability.

When assessing transitions from ISC to IUC, the panel found it was appropriate to transition based solely on patient requests (a patient-centered approach). However, patients must understand the potential risks of IUC, including increased infection risk and restraints on normal activity,^24,25^ so that their decision is informed.

The recommendations of the panel indicated that bladder scanners allow for data-driven patient management. This requires the ubiquitous presence of bladder scanners in hospitals. For an investment of several thousand dollars each, having scanners readily available can provide rapid, noninvasive diagnosis and prevent unnecessary catheterization.

Panel findings provided the numerical framework for our draft algorithm, which we then iteratively refined through qualitative interviews with local stakeholders. Honoring frontline clinicians’ request for specific cutoffs rather than numeric ranges required choosing somewhat arbitrary cutoffs (for volumes and timing) within the range of options deemed appropriate by the panel. This underlines the fact that the numerical choices in our algorithm are not the only reasonable options consistent with the panel data; thus, our results allow adaptation of the URECA to fit various patient populations and health system policies.

Future efforts could examine whether algorithms such as the URECA can minimize the harms of untreated UR and unnecessary urinary catheterization or whether unintended consequences arise from algorithm implementation. The algorithm was recently implemented in the Michigan Surgical Quality Collaborative as a tool in the 2023 SUCCESS quality improvement initiative in multiple participating Michigan hospitals to assess diagnosis and management of UR, measure patient outcomes regarding harms related to urinary catheter use, and provide qualitative data regarding implementation and feedback regarding the tool’s usability.

### Limitations

There are several caveats and limitations to this study. First, the RAND/UCLA Appropriateness Method panel included only 11 members, though this aligns with the formal methods^11^ and we sampled panelists from a range of institutions and practice types across the US to capture diverse perspectives. We also included urologists who regularly manage complex UR in addition to practitioners with general medical backgrounds.^26,27^ Second, although we systematically assessed the relevant literature, the evidence was not of optimal quality, lacking randomized clinical trials to guide appropriateness ratings. Since the literature indicates that most inpatient catheters are placed due to surgery,^25^ the qualitative critique of the algorithm focused on patients at risk for postoperative UR. Therefore, some of the practitioners interviewed had less expertise in the management of nonsurgical patients and the algorithm may not be optimized for the general medical population. Third, the panel and the URECA focused decision-making based on postvoid residual volumes and did not consider voided volumes or calculations of voiding efficiency, which can be relevant in patients with low bladder capacities.^28^ Future algorithm iterations could be adapted to include these parameters.

## Conclusions

This mixed-methods study defined clinically relevant guidance for the appropriateness of UR screening and management, developed an algorithm consistent with this guidance, and refined the algorithm through qualitative interviews with stakeholders. Given the prevalence of UR, algorithms such as the URECA have the potential to improve patient safety by reducing inappropriate catheterizations.

## Notes

Supplement 1. **eTable 1.** Articles Yielded by Systematic Search Provided to the RAND/UCLA Appropriateness Panel Members **eTable 2.** Cochairs and Panelists for the RAND/UCLA Appropriateness Panel **eTable 3** Representative Questions From the Pilot-Tested, Semistructured, Role-Specific Interview Guide **eTable 4.** Appropriateness Ratings for Monitoring of Urinary Retention After Catheter Removal **eTable 5.** Presumptive Treatment of Urinary Retention After Catheter Removal When a Bladder Scanner Is Unavailable **eTable 6.** Appropriateness Ratings for Treatment of Urinary Retention Based on Bladder Scan Volume **eTable 7.** Appropriateness Ratings for Transitioning From Intermittent to Indwelling Catheter for Urinary Retention **eTable 8.** Preoperative Strategies to Predict or Prevent Postoperative Urinary Retention **eFigure 1.** Overview of Multimethod Approach Applied to Generate Final Algorithm **eFigure 2.** Early Retention Algorithm Drafts **eFigure 3:** Final Urinary Retention Evaluation and Catheterization Algorithm (URECA)

Supplement 2.
Data Sharing Statement
