# Supplementary material for: Urinary Retention Evaluation and Catheterization Algorithm for Adult Inpatients
Source: JAMA Netw Open. 2024 Jul 16;7(7):e2422281. doi: 10.1001/jamanetworkopen.2024.22281 (PMC11252892; doi:10.1001/jamanetworkopen.2024.22281)
Supplement: Supplement 1. — eTable 1. Articles Yielded by Systematic Search Provided to the RAND/UCLA Appropriateness Panel Members eTable 2. Cochairs and Panelists for the RAND/UCLA Appropriateness Panel eTable 3 Representative Questions From the Pilot-Tested, Semistructured, Role-Specific Interview Guide eTable 4. Appropriateness Ratings for Monitoring of Urinary Retention After Catheter Removal eTable 5. Presumptive Treatment of Urinary Retention After Catheter Removal When a Bladder Scanner Is Unavailable eTable 6. Appropriateness Ratings for Treatment of Urinary Retention Based on Bladder Scan Volume eTable 7. Appropriateness Ratings for Transitioning From Intermittent to Indwelling Catheter for Urinary Retention eTable 8. Preoperative Strategies to Predict or Prevent Postoperative Urinary Retention eFigure 1. Overview of Multimethod Approach Applied to Generate Final Algorithm eFigure 2. Early Retention Algorithm Drafts eFigure 3: Final Urinary Retention Evaluation and Catheterization Algorithm (URECA) [file jamanetwopen-e2422281-s001.pdf]

Supplementary Online Content

Chrouser K, Fowler KE, Mann JD, et al. Urinary retention evaluation and catheterization algorithm for adult inpatients. *JAMA Netw Open*. 2024;7(7):e2422281. doi:10.1001/jamanetworkopen.2024.22281

eTable 1. Articles Yielded by Systematic Search Provided to the RAND/UCLA Appropriateness Panel Members
eTable 2. Cochairs and Panelists for the RAND/UCLA Appropriateness Panel
eTable 3 Representative Questions From the Pilot-Tested, Semistructured, Role-Specific Interview Guide
eTable 4. Appropriateness Ratings for Monitoring of Urinary Retention After Catheter Removal
eTable 5. Presumptive Treatment of Urinary Retention After Catheter Removal When a Bladder Scanner Is Unavailable
eTable 6. Appropriateness Ratings for Treatment of Urinary Retention Based on Bladder Scan Volume
eTable 7. Appropriateness Ratings for Transitioning From Intermittent to Indwelling Catheter for Urinary Retention
eTable 8. Preoperative Strategies to Predict or Prevent Postoperative Urinary Retention
eFigure 1. Overview of Multimethod Approach Applied to Generate Final Algorithm
eFigure 2. Early Retention Algorithm Drafts
eFigure 3: Final Urinary Retention Evaluation and Catheterization Algorithm (URECA)

This supplementary material has been provided by the authors to give readers additional information about their work.

© 2024 Chrouser K et al. *JAMA Network Open*.

eTable 1. Articles yielded by the systematic search that were provided to the RAND/UCLA Appropriateness Panel members

Article Category	References
"A" articles that specifically tested a feature of urinary retention protocol, such as accuracy of a bladder scanner to assess bladder volumes	1. Pedersen JF, Bartrum RJ, Grytter C. Residual urine determination by ultrasonic scanning. *Am J Roentgenol Radium Ther Nucl Med.* Oct 1975;125(2):474-478.<br>2. Ravichandran G, Fellows GJ. The accuracy of a hand-held real time ultrasound scanner for estimating bladder volume. *British journal of urology.* 1983;55(1):25-27.<br>3. Rosseland LA, Stubhaug A, Breivik H. Detecting postoperative urinary retention with an ultrasound scanner. *Acta Anaesthesiologica Scandinavica.* 2002;46(3):279-282.<br>4. Wilson M. Ultrasound assessment of bladder volume: is it valid after delivery? *The Australian & New Zealand journal of obstetrics & gynaecology.* 2003;43(6):453-456.<br>5. Crouch N, O'Brien P, O'Sullivan C, Cutner A. Intrapartum ultrasound estimation of total bladder volume. *BJOG : an international journal of obstetrics and gynaecology.* 2004;111(2):103-108.<br>6. Rosseland LA, Bentsen G, Hopp E, Refsum S, Breivik H. Monitoring urinary bladder volume and detecting post-operative urinary retention in children with an ultrasound scanner. *Acta anaesthesiologica Scandinavica.* 2005;49(10):1456-1459.<br>7. Cederkvist HR, Rosseland LA. Reliability of an automatic ultrasound system for detecting postpartum urinary retention after vaginal birth. *Acta obstetricia et gynecologica Scandinavica.* 2007;86(10):1251-1255.<br>8. Park YH, Ku JH, Oh S-J. Accuracy of post-void residual urine volume measurement using a portable ultrasound bladder scanner with real-time pre-scan imaging. *Neurourology and urodynamics.* 2011;30(3):335-338.

© 2024 Chrouser K et al. *JAMA Network Open*.

**Article Category**	**References**
"B" articles that did not specifically test a urinary retention protocol as part of the intervention but included a detailed urinary retention protocol for review. These "B" articles are included to demonstrate the variety of urinary retention protocols in use.	1. Moore DA, Edwards K. Using a portable bladder scan to reduce the incidence of nosocomial urinary tract infections. *MEDSURG Nursing.* 1997;6(1):39-43.<br>2. Luger TJ, Garoscio I, Rehder P, Oberladstatter J, Voelckel W. Management of temporary urinary retention after arthroscopic knee surgery in low-dose spinal anesthesia: development of a simple algorithm. *Arch Orthop Trauma Surg.* Jun 2008;128(6):607-612.<br>3. Boyer DR, Steltzer N, Larrabee JH. Using the best evidence to change practice. Implementation of an evidence-based bladder scanner protocol. *Journal of Nursing Care Quality.* 2009 Jan-Mar 2009;24(1):10-16.<br>4. Brouwer T, Kalkman C, Moons C. Post operative urinary retention: Can we do better? *European Journal of Anaesthesiology.* 2009;26:19.<br>5. Shadle B, Barbaro C, Waxman K, Connor S, Von Dollen K. Predictors of postoperative urinary retention. *American Surgeon.* 2009;75(10):922-924.<br>6. Balderi T, Mistraletti G, D'Angelo E, Carli F. Incidence of postoperative urinary retention (POUR) after joint arthroplasty and management using ultrasound-guided bladder catheterization. *Minerva Anestesiologica.* 2011;77(11):1051-1057.<br>7. Cutright J. The effect of the bladder scanner policy on the number of urinary catheters inserted. *Journal of wound, ostomy, and continence nursing : official publication of The Wound, Ostomy and Continence Nurses Society / WOCN.* 2011;38(1):71-76.<br>8. Mateu N, Perez N, Tantina S, Benavent R. Avoiding urinary catheterisation in acute geriatric service at the granollers general hospital using a bladder scan. *European Geriatric Medicine.* 2011;2:S195.<br>9. Kim HJ, Chun MH, Han EY, Yi JH, Kim DK. The utility of a bladder scan protocol using a portable ultrasonographic device in subacute stroke patients. *Disability and rehabilitation.* 2012;34(6):486-490.<br>10. Rhyne L, Potter T, Headrick JR. Is It safe to remove urinary catheters in general thoracic surgery patients receiving thoracic epidural analgesia within the first 24 hours? *Chest.* 2012;142(4).<br>11. Uberoi V, Calixte N, Orlando R, Lerner L. A strategy to reduce Foley days, post-operative urinary retention, and catheter-associated urinary tract infections. *Journal of Urology.* 2012;187(4):e111-e112.<br>12. Zaouter C, Wuethrich P, Miccoli M, Carli F. Early removal of urinary catheter leads to greater post-void residuals in patients with thoracic epidural. *Acta Anaesthesiologica Scandinavica.* Sep 2012;56(8):1020-1025.<br>13. Antonescu I, Baldini G, Watson D, et al. Impact of a bladder scan protocol on discharge efficiency within a care pathway for

© 2024 Chrouser K et al. *JAMA Network Open*.

	ambulatory inguinal herniorraphy. *Surgical endoscopy.* 2013;27(12):4711-4720.<br>14. Tripepi-Bova KA, Sun Z, Mason D, Albert NM. Early Removal of Urinary Catheters in Patients With Thoracic Epidural Catheters. *Journal of Nursing Care Quality.* 2013;28(4):340-344.<br>15. Lai KY, Chan WK, Ho WF, Leung CS, Li YK. Application of bladder scan in ambulatory management protocol for acute urinary retention with presumed diagnosis of benign prostatic hypertrophy. *Hong Kong Journal of Emergency Medicine.* 2014;21(5):300-307.

**Article Category**	**References**
"C" articles include literature available in intervention studies that assessed AUA or IPSS symptom scores as predictors of post-operative urinary retention, in a variety of different common surgical procedures.	1. Elkhodair S, Parmar HV, Vanwaeyenbergh J. The role of the IPSS (International Prostate Symptoms Score) in predicting acute retention of urine in patients undergoing major joint arthroplasty. *The Surgeon.* 4// 2005;3(2):63-65.<br>2. Cronin JJ, Shannon FJ, Bale E, Quinlan W. Prediction of post-operative urinary retention in hip and knee arthroplasty in a male population. *Eur J Orthop Surg Traumatol.* 2007/01/01 2007;17(1):47-50.

© 2024 Chrouser K et al. *JAMA Network Open*.

eTable 2. Panel Co-Chairs and Panelists for the RAND/UCLA Appropriateness Panel

Co-Chairs / Panel Leaders (not-voting)

Name	Title*	Affiliation*	RAND/UCLA Appropriateness Method Experience
Jennifer Meddings, MD, MSc	Assistant Professor	University of Michigan, Ann Arbor, MI	Experience conducting multiple RAND/UCLA appropriateness panels
Steven Bernstein, MD, MPH	Professor	University of Michigan, Ann Arbor, MI	Co-author of *The RAND/UCLA appropriateness method user's manual*
Ted Skolarus, MD,	Assistant Professor	University of Michigan, Ann Arbor, MI	Experience participating in and conducting multiple RAND/UCLA appropriateness panels

Panelists**

Name	Title*	Affiliation*	Specialty
Michael Balk, RN	Operating Room Circulating Nurse	Mercy Health Saint Mary's Hospital, Grand Rapids, MI	Nursing
Donald R. Bodner, MD	Professor of Urology	Louis Stokes Cleveland VA Medical Center, Cleveland, OH<br>Case Western Reserve University School of Medicine, Cleveland, OH	Urology
Sansern Borirakchanyavat, MD	Chief of Urology	Southern California Permanente Medical Group, Panorama City, CA	Urology
Bruce L. Jacobs, MD, MPH	Assistant Professor	University of Pittsburgh School of Medicine, Pittsburgh, PA	Urology
John T. Leppert, MD, MS	Assistant Professor	Stanford University, Stanford, CA;<br>VA Palo Alto Health Care System, Palo Alto, CA	Urology
Daniel J. Morgan, MD, MS	Associate Professor; Hospital Epidemiologist	University of Maryland, Baltimore, MD;<br>VA Maryland Healthcare System, Baltimore, MD	Infectious Diseases
Michael C. Risk, MD, PhD	Assistant Professor	University of Minnesota, Minneapolis, MN;<br>VA Minneapolis Health Care System, Minneapolis, MN	Urology
Andrea Starnes, RN	Infectious Diseases Nurse Case Manager	VA Ann Arbor Healthcare System, Ann Arbor, MI	Infectious Diseases; Nursing

© 2024 Chrouser K et al. *JAMA Network Open*.

Seth A. Strope, MD, MPH	Assistant Professor	Washington University School of Medicine, St. Louis, MO	Urology
Jonathan N. Warner, MD	Assistant Professor	University of Michigan, Ann Arbor, MI	Urology
John T. Wei, MD, MS	Professor	University of Michigan, Ann Arbor, MI	Urology

*At time of panel participation

******Panelists selected by co-chairs based on clinical expertise and geographic diversity. Panelists were invited to participate via email. Panelists were allowed to suggest other members for the panel. These suggestions were then reviewed by co-chairs before deciding whether to officially invite the clinician.

© 2024 Chrouser K et al. *JAMA Network Open*.

eTable 3. Representative Questions from the Pilot-Tested, Semi-Structured Role-Specific Interview Guide

URINARY RETENTION ALGORITHM QUESTIONS
What protocols are in place for managing post-op urinary retention?<br>[If none ask]: What protocols or guidelines would you find most helpful?<br>What (content) would that include? Key decision points?<br>When to re-insert Foley? When to consult with urology?<br>Would you like protocols on paper checklists?<br>In EMR, on a website, on a phone app?
We are considering developing a protocol to help manage and diagnose post-op urinary retention. Are you involved in the management of post-op urinary retention?<br>[If yes ask]: We would welcome your opinions on a draft protocol we are considering:<br>What do you think of this draft protocol?<br>What do you like about it? How could we improve it?<br>Where should this algorithm be found? For example, within the EMR or posted in the PACU?<br>What changes would be needed to make a protocol work effectively in your setting?

© 2024 Chrouser K et al. *JAMA Network Open*.

eTable 4. Appropriateness Ratings for Monitoring of Urinary Retention after Catheter Removal

<table>
<tr><th rowspan="2">Clinical Scenario:
Lack of urine output in post-operative patient or medical inpatient</th><th colspan="2">Evaluation Using Bladder Scanner</th><th colspan="2">Evaluation Using Intermittent Straight Cath (ISC)
(instead of scanning)</th><th colspan="2">Evaluation Using Indwelling Urinary Catheter (IUC) Placement
(instead of scanning)</th></tr>
<tr><th>with symptoms</th><th>without symptoms</th><th>with symptoms</th><th>without symptoms</th><th>with symptoms</th><th>without symptoms</th></tr>
<tr><td>No urine output <1 hour</td><td rowspan="7">Appropriate</td><td rowspan="2">Inappropriate</td><td rowspan="7" colspan="4">Inappropriate</td></tr>
<tr><td>No urine output 1 to <2 hours</td></tr>
<tr><td>No urine output 2 to <3 hours</td><td>Uncertain</td></tr>
<tr><td>No urine output 3 to <4 hours</td><td rowspan="4">Appropriate</td></tr>
<tr><td>No urine output 4 to <5 hours</td></tr>
<tr><td>No urine output 5 to <6 hours</td></tr>
<tr><td>No urine output > 6 hours</td></tr>
</table>

© 2024 Chrouser K et al. *JAMA Network Open*.

eTable 5. Presumptive Treatment of Urinary Retention after Catheter Removal When a Bladder Scanner is Unavailable

<table>
<tr><th rowspan="2">Clinical Scenario: Lack of urine output in post-operative patient or medical patient</th><th colspan="2">Intermittent Catheterization</th><th colspan="2">Indwelling Catheter Placement Overnight</th></tr>
<tr><th>with symptoms</th><th>without symptoms</th><th>with symptoms</th><th>without symptoms</th></tr>
<tr><td>No urine output <1 hour</td><td>Inappropriate</td><td rowspan="4">Inappropriate</td><td rowspan="3">Inappropriate</td><td rowspan="5">Inappropriate</td></tr>
<tr><td>No urine output 1 to <2 hours</td><td rowspan="2">Uncertain</td></tr>
<tr><td>No urine output 2 to <3 hours</td></tr>
<tr><td>No urine output 3 to <4 hours</td><td rowspan="4">Appropriate</td><td rowspan="2">Uncertain</td></tr>
<tr><td>No urine output 4 to <5 hours</td><td>Uncertain</td></tr>
<tr><td>No urine output 5 to <6 hours</td><td rowspan="2">Appropriate</td><td rowspan="2">Appropriate</td><td rowspan="2">Uncertain</td></tr>
<tr><td>No urine output > 6 hours</td></tr>
</table>

© 2024 Chrouser K et al. *JAMA Network Open*.

eTable 6. Appropriateness Ratings for Treatment of Urinary Retention Based on Bladder Scan Volume

<table>
<tr><th rowspan="2">Clinical Scenario: Post void scanned volumes</th><th colspan="2">Intermittent Straight Catheterization (ISC)</th><th colspan="2">Indwelling Urinary Catheter (IUC) Placement Overnight</th></tr>
<tr><th>with symptoms</th><th>without symptoms</th><th>with symptoms</th><th>without symptoms</th></tr>
<tr><td><100 ml</td><td>Inappropriate</td><td rowspan="4">Inappropriate</td><td rowspan="3">Inappropriate</td><td rowspan="4">Inappropriate</td></tr>
<tr><td>100-199 ml</td><td rowspan="2">Uncertain</td></tr>
<tr><td>200-299 ml</td></tr>
<tr><td>300-399 ml</td><td rowspan="3">Appropriate</td><td>Uncertain</td></tr>
<tr><td>400-499 ml</td><td>Uncertain</td><td rowspan="2">Appropriate</td><td>Uncertain</td></tr>
<tr><td>500 ml and up</td><td>Appropriate</td><td>Appropriate</td></tr>
</table>

© 2024 Chrouser K et al. *JAMA Network Open*.

eTable 7. Appropriateness Ratings for Transitioning from Intermittent to Indwelling Catheter for Urinary Retention

Clinical Scenarios	Transition from ISC to IUC
Patient requests indwelling catheter before any intermittent straight catheterization (ISC) attempted	Appropriate
Patient requests indwelling catheter after 1 ISC	
Patient requests indwelling catheter after 2 or more ISCs	
ISC needed once daily for 2 or more days	Inappropriate
ISC needed 2 times in 24 hours	
ISC needed 3 times in 24 hours	
ISC needed 4 times in 24 hours	
ISC needed bid for 2 or more days	
ISC needed >bid for 2 or more days	
ISC needed more often than q 4 hours	Appropriate
ISC output >500ml q 4 hours	
Initial ISC output <500ml	Inappropriate
Initial ISC output >500ml	Uncertain

© 2024 Chrouser K et al. *JAMA Network Open*.

eTable 8. Pre-Operative Strategies to Predict or Prevent Post-Operative Urinary Retention

<table>
<tr><th>Clinical Scenarios:</th><th>Assessment/ Treatment</th><th>Indwelling Catheter Placement</th><th>First Trial of Void</th></tr>
<tr><td colspan="4">Conducting the following pre-operative assessments ROUTINELY for adult male patients</td></tr>
<tr><td>AUA/IPSS to inform management of post-operative UR</td><td rowspan="3">Uncertain</td><td></td><td></td></tr>
<tr><td>AUA/IPSS to inform the need for IUC in the operating room and timing of removal post-operatively</td><td></td><td></td></tr>
<tr><td>Post void residual by bladder scanner in a patient with a history of prior UR</td><td></td><td></td></tr>
<tr><td>Post void residual by bladder scanner in a patient who reports having difficulty voiding</td><td>Inappropriate</td><td></td><td></td></tr>
<tr><td>Placing IUC ROUTINELY in the operating room in an adult male with any prior history of UR (assuming IUC otherwise not needed)</td><td></td><td>Inappropriate</td><td></td></tr>
<tr><td colspan="4">Conducting the FIRST trial of void on this day in an adult male post-operative patient with any prior history of UR</td></tr>
<tr><td>Post-operative day #0</td><td></td><td></td><td rowspan="2">Appropriate</td></tr>
<tr><td>Post-operative day #1</td><td></td><td></td></tr>
<tr><td>Post-operative day #2</td><td></td><td></td><td rowspan="3">Inappropriate</td></tr>
<tr><td>Post-operative day #3</td><td></td><td></td></tr>
<tr><td>Post-operative day #4 or later</td><td></td><td></td></tr>
<tr><td>Initiating alpha blocker medication (e.g., doxazosin, terazosin, or tamsulosin), if not contra-indicated, to PREVENT acute UR in a post-operative adult male or female patient with a history of prior post-operative UR and not currently being treated for UR</td><td>Uncertain</td><td></td><td></td></tr>
</table>

AUA: American Urological Association Symptom Score; IPSS: International Prostate Symptom Score; UR Urinary Retention; IUC: Indwelling Urinary Catheter.

© 2024 Chrouser K et al. *JAMA Network Open*.

eFigure 1. Overview of multi-method approach applied to generate final algorithm

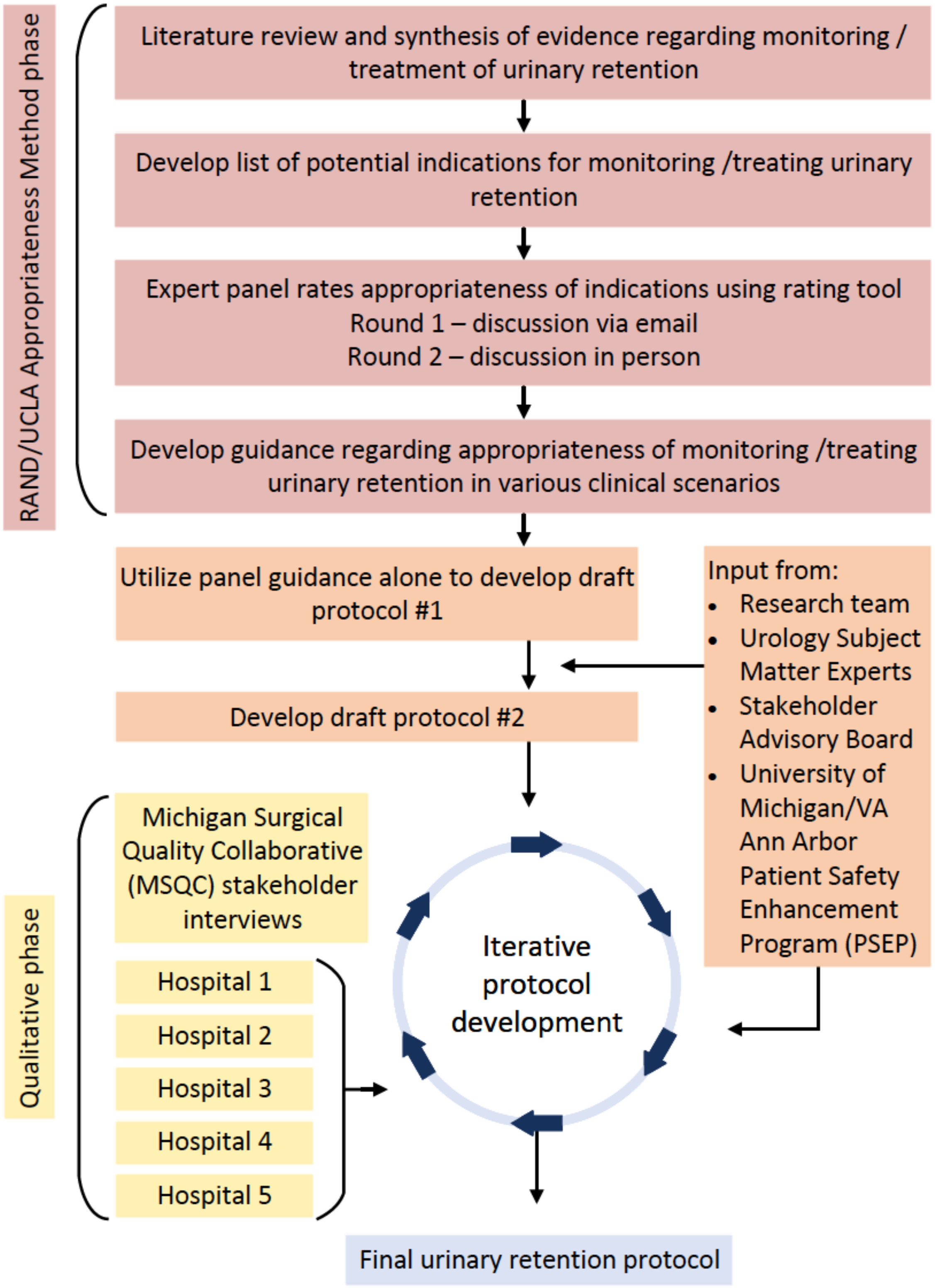

© 2024 Chrouser K et al. *JAMA Network Open*.

eFigure 2. Early Drafts of the Urinary Retention Evaluation and Catheterization Algorithm (URECA)

Draft Algorithm #1 Retention Evaluation and Management

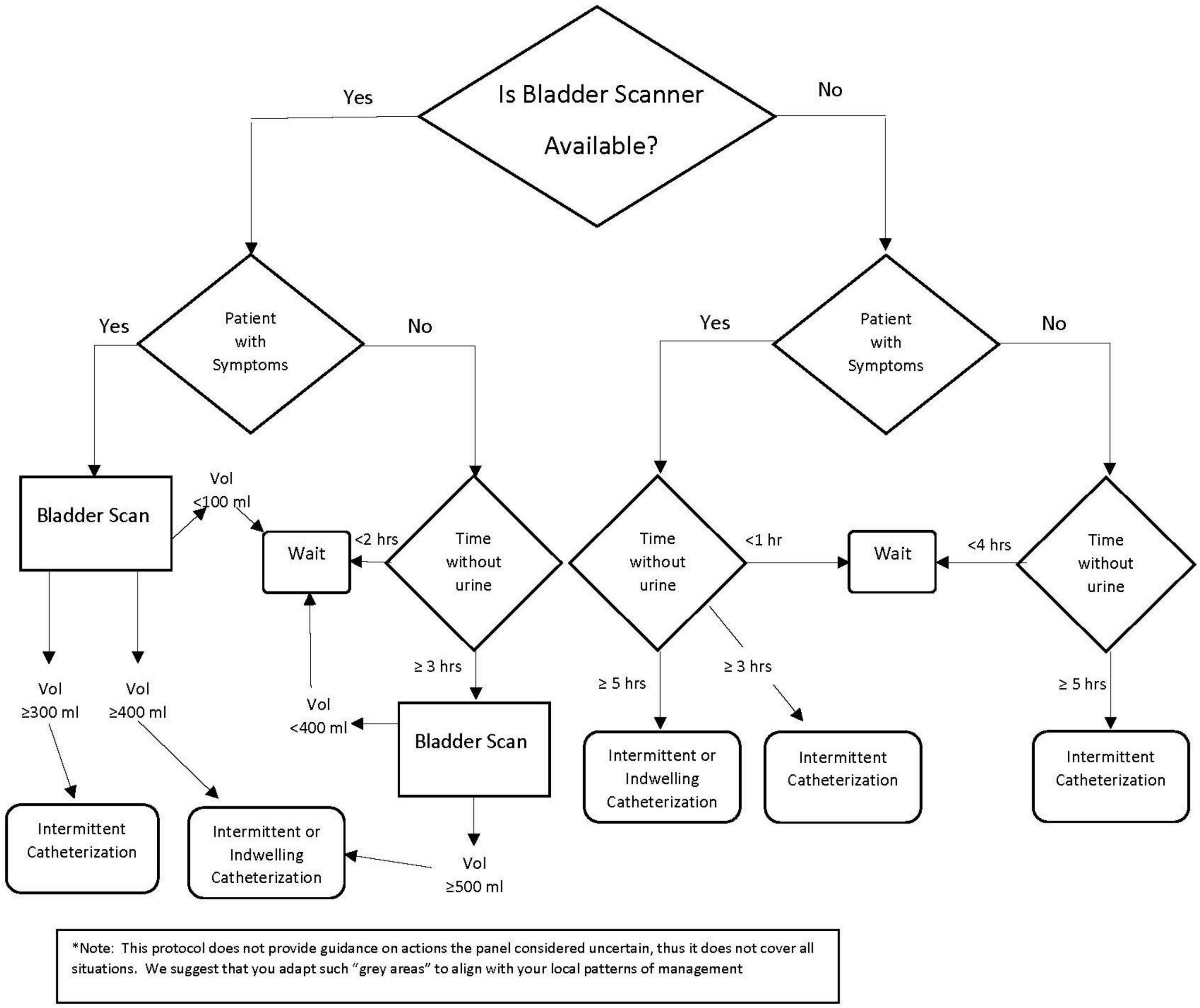

© 2024 Chrouser K et al. *JAMA Network Open*.

Draft Algorithm #2 Retention Evaluation and Management

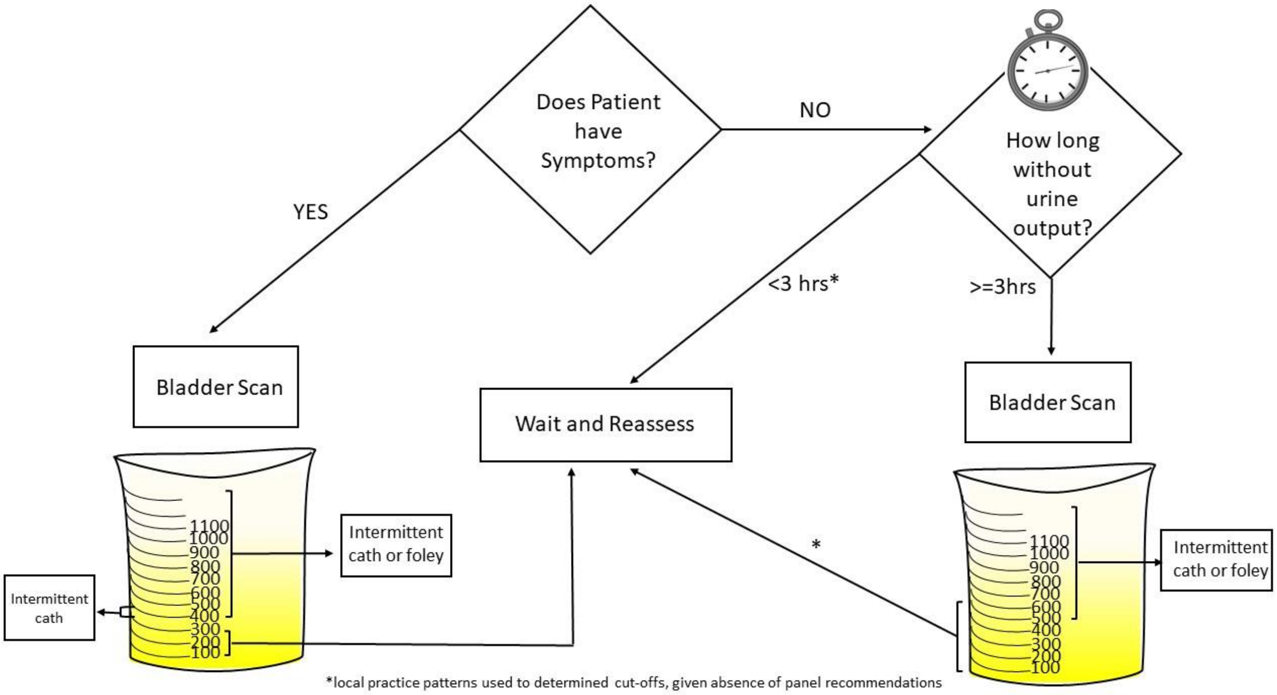

© 2024 Chrouser K et al. *JAMA Network Open*.

eFigure 3: Final Urinary Retention Evaluation and Catheterization Algorithm (URECA) (Full color version with icons)

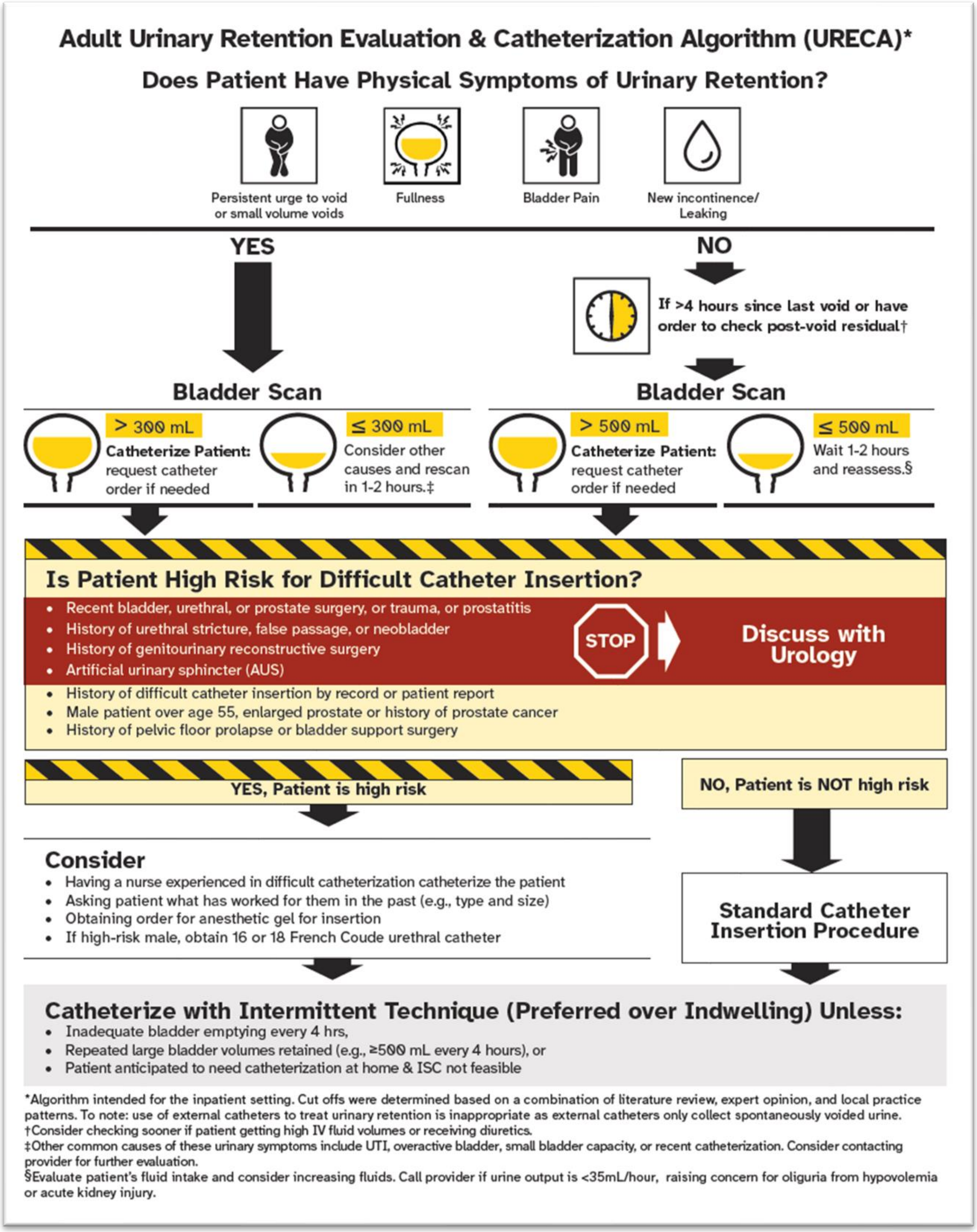

© 2024 Chrouser K et al. *JAMA Network Open*.
