# Supplementary material for: Urinary Retention Evaluation and Catheterization Algorithm for Adult Inpatients
Source: JAMA Netw Open. 2024 Jul 16;7(7):e2422281. doi: 10.1001/jamanetworkopen.2024.22281 (PMC11252892; doi:10.1001/jamanetworkopen.2024.22281)
Supplement: Supplement 2. — Data Sharing Statement [file jamanetwopen-e2422281-s002.pdf]

Data Sharing Statement

Chrouser. Urinary Retention Evaluation and Catheterization Algorithm for Adult Inpatients.
JAMA Netw Open. Published July 16, 2024. doi:10.1001/jamanetworkopen.2024.22281

Data
Data available: Yes
Data types: Data (not involving human participants)
How to access data: Raw data from the RAND/UCLA Appropriateness panels will be provided upon request to the corresponding author, Dr. Chrouser (chrouser@med.umich.edu).
When available: With publication

Supporting Documents
Document types: None

Additional Information
Who can access the data: Researchers whose proposed use of the data has been approved.
Types of analyses: For any purpose.
Mechanisms of data availability: With investigator support.
